# Construction of finite element model and stress analysis of anterior cruciate ligament tibial insertion

**DOI:** 10.12669/pjms.313.7208

**Published:** 2015

**Authors:** Can Dai, Liu Yang, Lin Guo, Fuyou Wang, Jingyue Gou, Zhilong Deng

**Affiliations:** 1 Can Dai, MM. Center for Joint Surgery, Southwest Hospital, Third Military Medical University, Chongqing, 400038, China; 2 Liu Yang, MD. Center for Joint Surgery, Southwest Hospital, Third Military Medical University, Chongqing, 400038, China; 3 Lin Guo, MD. Center for Joint Surgery, Southwest Hospital, Third Military Medical University, Chongqing, 400038, China; 4 Fuyou Wang, MD. Center for Joint Surgery, Southwest Hospital, Third Military Medical University, Chongqing, 400038, China; 5 Jingyue Gou, MB. Department of orthopedics, Emergency Medical Center, Chongqing, 400014, China; 6 Zhilong Deng, MD. Department of orthopedics, Emergency Medical Center, Chongqing, 400014, China

**Keywords:** Anterior Cruciate Ligament, Insertion, Stress, Finite element

## Abstract

**Objective:**

The aim of the present study was to develop a more realistic finite element (FE) model of the human anterior cruciate ligament (ACL) tibial insertion and to analyze the stress distribution in the ACL internal fibers under load.

**Methods:**

The ACL tibial insertions were processed histologically. With Photoshop software, digital images taken from the histological slides were collaged, contour lines were drawn, and different gray values were filled based on the structure. The data were exported to Amira software and saved as “.hmascii” file. This document was imported into HyperMesh software. The solid mesh model generated using HyperMesh software was imported into Abaqus software. The material properties were introduced, boundary conditions were set, and load was added to carry out the FE analysis.

**Results:**

The stress distribution of the ACL internal fibers was uneven. The lowest stress could be observed in the ACL lateral fibers under tensile and shear load.

**Conclusion:**

The establishment of ACL tibial insertion FE model and mechanical analysis could reveal the stress distribution in the ACL internal fibers under load. There was greater load carrying capacity in the ACL lateral fibers which could sustain greater tensile and shear forces.

## INTRODUCTION

Anterior cruciate ligament (ACL) has been widely studied to analyze its function in joint stability and load transmission by in vivo and in vitro experiments or numerical simulations.^1^-^4^ The finite element (FE) model of the ACL can provide some useful information otherwise difficult to obtain from experiments. In previous studies, many simple loading conditions have been tested to analyze the stress distribution of ACL in the kinematic characteristics of human knee joint.^5^-^8^ In the present study, the authors tried to analyze the stress distribution of ACL focusing on the mechanical characteristics of the ACL tibial insertion tissues.

An ACL tibial insertion consists of four distinct tissue layers of transition, namely ligaments, uncalcified fibrocartilage (UF), calcified fibrocartilage (CF), and subchondral bone. The region-dependent matrix organization and the interface subdivision into uncalcified and calcified regions caused a gradual increase in mechanical properties across the interface regions and minimized the stress levels, enabling effective load transfer from ligament to bone.^9^ Each of inserted tissues played a different role in the force transmission from ACL to tibia, which would obviously influence the stress distribution of the ACL internal fibers. The aim of the present study was to develop a more realistic FE model of the human ACL tibial insertion and to analyze the stress distribution in the ACL internal fibers under load.

## METHODS

### Specimen Preparation

The right knee joint was obtained from our hospital’s Bone Tissue Engineering Center (body height was 169 cm and body weight was 61 kg). The subject had died in a traffic accident and was judged as having no signs of gross bony deformity, previous fractures, or degenerative diseases in the knee by X-ray and magnetic resonance imaging (MRI). The ACL was not divided into the anteromedial (AM) and posterolateral (PL) bundles macroscopically. The ACL tibial insertion was identified and removed from the fresh knee joint within 48 hour after subject’s death. The sample contained the distal part of the ligament (4mm) and its insertion together with the bone (4mm). The block was fixed in 10% neutral buffered formalin, decalcified with 5% nitric acid, dehydrated through a graded alcohol series, cleared in xylene, and embedded in paraffin wax. Serial 5-μm thick transverse sections were cut and mounted on glass slides at 50-μm intervals. The present study complied with the Helsinki Declaration.

### Two-Dimensional Image Processing

**Fig.1:**
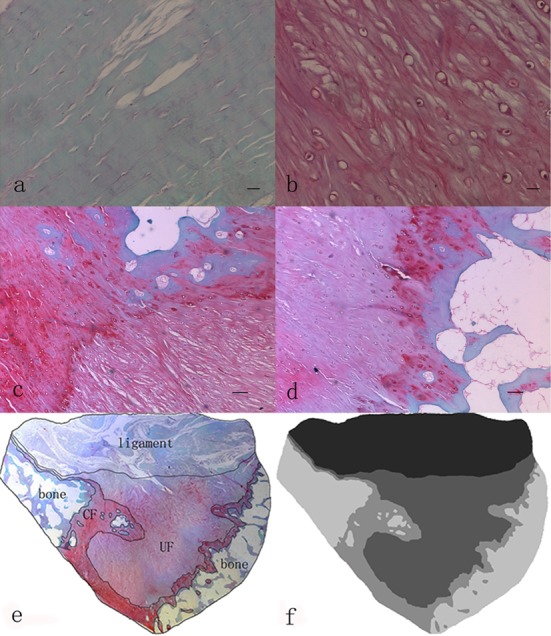
(a) There were fusiform and spindle-shape fibroblasts and a high-density collagen bundles in ligaments, which was stained as green. Safranin O/fast green staining. Scale bars = 0.02 mm. (b) The fibrocartilage cells were round and ovoid in the UF region, and the area was stained as red. Safranin O/fast green staining. Scale bars = 0.02 mm. (c) and (d) The junction between the CF and the subchondral bone was highly irregular in contrast to the junction between UF and CF. There was a jigsaw-like interlocking of CF pieces and bone. Safranin O/fast green staining. Scale bars = 0.1 mm. (e) The contour lines of four zones were drawn manually. (f) The two-dimensional images were filled with different gray values.

The sections were stained with hematoxylin for 3 minutes, differentiated in 1% acid alcohol for 15 s, stained again with 0.02% aqueous fast green for 3 minutes, and counterstained with 0.1% Safranin O for 3 minutes, as described previously.^10^ Finally, the sections were dehydrated through a graded alcohol series, cleared in xylene, and mounted onto glass slides using neutral gum. Each stained section was observed under light microscope and photographed using a photomicroscope equipped with a charge-coupled device video camera. With the use of Photoshop 8.0 software (Adobe Company, San Jose, CA, USA), the serial digital images taken from each transverse histological slide were automatically collaged to obtain the total image. The four zones including fibrous tissue, UF, CF, and subchondral bone, were distinguished according to their histomorphology and the staining. The fibrous tissue region was characterized by the presence of spindle-shaped fibroblasts lying between the collagen fibers, and this region was stained as green in color (Fig.1a). The UF range was estimated from the tidemark to the furthest recognizable chondrocyte within the ligament, as described previously,^11^ and this area was stained in red through Safranin O staining (Fig.1b). The CF zone was defined as the area between the tidemark and the subchondral bone and the subchondral bone zone had organized lamellar bones. The bone zone was stained blue or green (Fig.1c and Fig.1d). The contour lines of four zones were drawn manually (Fig. 1e), and then the two-dimensional images were filled with different gray values manually using the Photoshop 8.0 software (Fig.1f).

### FE Model and Stress Analysis

**Fig.2:**
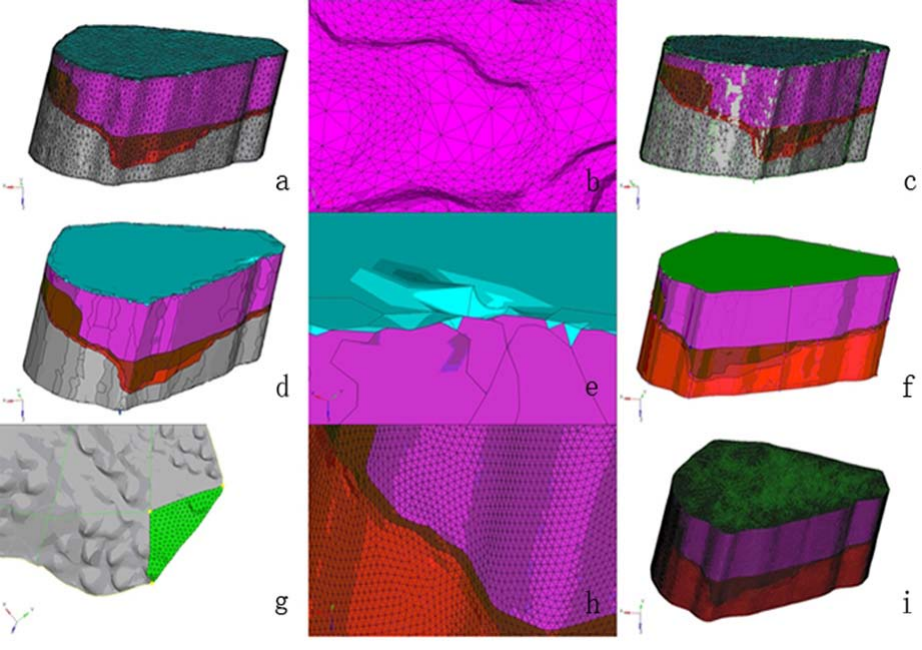
(a) The .hmascii file appeared an originally enveloped mesh model in HyperMesh software. (b) The original meshes sizes were variance. (c) The high-order surfaces which matched originally enveloped meshes were generated. (d) The originally enveloped meshes were deleted. (e) The high-order surfaces had many folds. (f) The high-order surfaces had been smoothed to get rid of the folds. (g) The model was filled with tetrahedron elements manually. (h) The meshes filled manually were a consistent size. (i) A solid mesh model was obtained.

All the transverse gray images were fed into Amira 5.2.0 software (VSG Company, Richmond VIC, Australia) and saved as “.hmascii” file. The .hmascii file was imported into HyperMesh 12.0 software (Altair Company, Troy, MI, USA) and appeared an originally enveloped mesh model of four segments. The original meshes sizes were variance. After the high-order surfaces which matched originally enveloped meshes were generated, the originally enveloped meshes were deleted. There were many folds in the high-order surfaces, so that it had been smoothed. The segments were filled with tetrahedron elements which were linked by nodes, and then a solid mesh model was obtained (Fig.2). The solid mesh model generated using HyperMesh 12.0 was imported into Abaqus 6.10 software (Dassault Systemes Simulia Company, Providence, RI, USA). Different material properties were introduced in various segments (Table-I). The material constants were obtained from the literature.^7^,^12^ The bottom of the bone completely fixed in the model. The tensile force (134 N) was loaded uniformly onto the proximal part of the ligament in the direction of long axis of ACL. The shear force (134 N) was loaded uniformly on all four directions, likely from anterior to posterior, posterior to anterior, medial to lateral, and lateral to medial.

**Table-I:** Material properties of each type of tissues in the current model (E: Young’s modulus; µ: Poisson’s ratio)

Material	Constitutive model	Model parameters
Ligament	Hyperelastic	C10=2.951 MPa
		C20=16.74693 MPa
		C30=63.3592 MPa
		C01=-1.49 MPa
Uncalcified fibrocartilage	Isotropic elastic	E=572 MPa, µ=0.432
Calcified fibrocartilage	Isotropic elastic	E=976 MPa, µ=0.366
Subchondral bone	Isotropic elastic	E=2780 MPa, µ=0.300

## RESULTS

### Model Establishment

A FE model of tibial insertion involving the ligament, UF, CF, and subchondral bone was established and developed using the HyperMesh 12.0 and Abaqus 6.10 software’s. The ligament segment contained 53,567 nodes and 249,959 elements. The UF segment contained 50,879 nodes and 233,765 elements. The CF segment contained 29,757 nodes and 111,759 elements. The subchondral bone segment contained 69,303 nodes and 330,769 elements.

### Tensile Load

**Fig.3:**
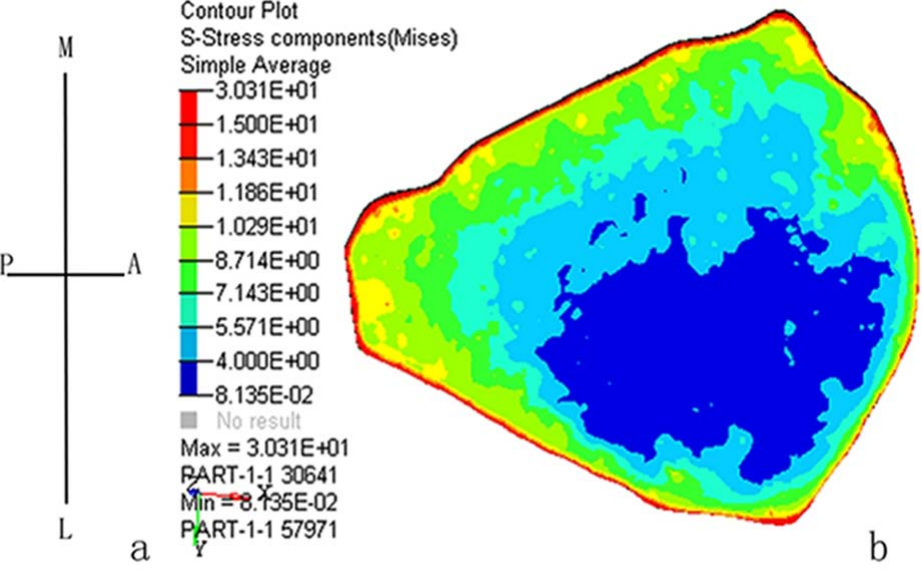
(a) Schematic drawing of the proximal insertion perspective. (A, anterior; P, posterior; M, medial; L, lateral). (b) Stress nephogram indicated that the ACL internal fibers tensile stress was distributed unevenly. The lowest von Mises stress was seen in the ACL lateral fibers.

A stress nephogram indicated that the ACL internal fibers tensile stress was distributed unevenly. The lowest von Mises stress was seen in the ACL lateral fibers (0.081-4 MPa). The stress was gradually increased towards radial direction. The von Mises stress was higher in ACL medial fibers (4-8.7 MPa) than in ACL lateral fibers (Fig.3).

### Shear Load

**Fig.4:**
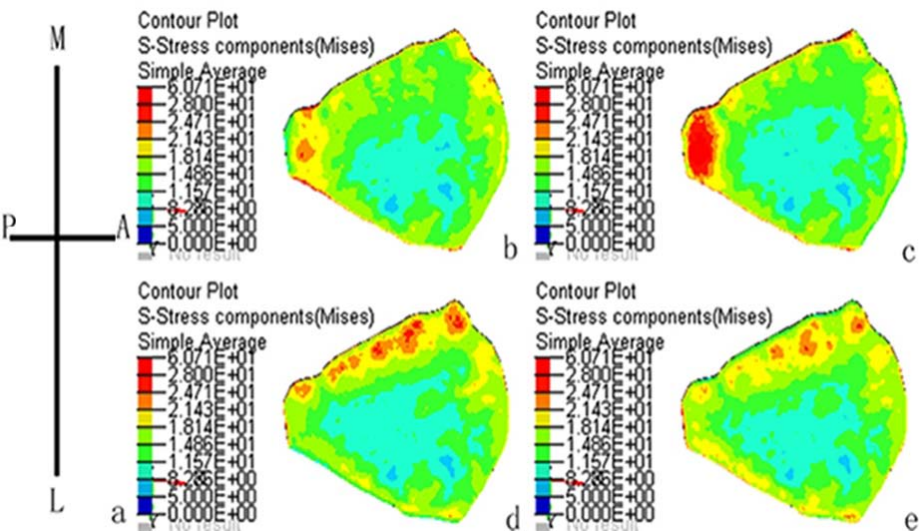
Stress nephogram indicated that the ACL internal fibers shear stress was distributed unevenly. The stress nephograms suggested that the lowest von Mises stress was seen in the ACL lateral fibers. (a) Schematic drawing of the proximal insertion perspective. (A, anterior; P, posterior; M, medial; L, lateral). The shear force was loaded from (b) anterior to posterior direction, (c) posterior to anterior direction, (d) medial to lateral direction and (e) lateral to medial direction.

A stress nephogram indicated that the ACL internal fibers shear stress was distributed unevenly. Under the anterior-posterior direction shear load, stress nephograms suggested that the lowest von Mises stress was seen in the ACL lateral fibers (5-11.6 MPa). The stress was gradually increased towards radial direction, and the ACL medial fibers stress value reached 11.6-18.1 MPa. The highest stress was found in the posterior region (18.1-28 MPa). Under the medial-lateral direction shear load, the lowest von Mises stress was seen in the ACL lateral fibers (5-11.6 MPa). The stress was gradually increased towards radial direction. Multiple stress concentration areas were formed in the medial region, and the von Mises stress value reached 18.1-28 MPa (Fig.4).

## DISCUSSION

In the present study, the authors have focused on three aspects on considering the available data from previous reports. Firstly, different modeling data differ substantially in modeling complex contours, and it directly influences the quality of geometric models. Traditional geometric modeling methods of biological tissues including frozen sectioning, MRI scanning, direct modeling, and three-dimensional laser scanning methods could not accurately distinguish the four distinct tissues, especially the ligament, UF, and CF. In the present study, the four distinct tissues were distinguished by histological method. The precise identification of the tissues in all two-dimensional images would ensure the geometric accuracy of the FE model.

Secondly, in the present study, the ACL tibial insertion was regarded as a functionally graded material with different elastic moduli. The mechanical properties of fibrocartilage (UF and CF) differed from ligament fibers. The presence of fibrocartilage between the ligament and the subchondral bone suggested a need for gradual change in the mechanical properties between the hard and soft tissues. This would dissipate stress concentration at the bony interface by promoting a gradual bending of collagen fibers. The existence of UF zone may be related to the shear stress and the CF zone may be related to the tensile stress of the tendon or ligament.^13^ In the present study, the authors viewed each tissue as an independent element and introduced different material properties in the FE model. It would reflect the stress distribution of the ACL internal fibers more accurately under loading conditions. In defining the material properties of ligament, the polynomial strain potential energy function model was used to simulate the ligament material, and the ligament was defined with the parameters of the three-order polynomial, and thus vividly reflecting ACL hyperelastic characteristics.^7^ The isotropic elastic model was used to simulate the other three tissues material including UF, CF, and subchondral bone at the ACL insertion. The material properties of these tissues have been reported in the literature.^12^

Finally, the kinematic characteristics of the knee joint such as tibial anterior/posterior translation, varus/valgus, and external/internal rotation were not defined in the present study. Regardless of kinematic state of knee joint, tensile and shear forces were directly loaded to tibial insertion in the FE model to analyze the stress distribution of the ACL internal fibers. The ACL played a key role against the anterior tibial force, which was regarded as 134 N by mechanical tests in previous studies.^5^,^8^ Hence, in the present study, the same tensile and shear force (134 N) was used in the FE model. The results revealed the lowest stress distribution in the ACL lateral fibers under tensile and shear load. It meant that there was greater load carrying capacity in ACL lateral fibers which could sustain greater tensile and shear forces and they played a more important role in the knee joint movement.

Although only one subject was used in this study, it was aimed to develop a more realistic FE model of the human ACL tibial insertion and to analyze the stress distribution in the ACL internal fibers under shear and tensile load. This goal was accomplished with the methodology developed in this study. Meanwhile, the classical mechanical testing methods were challenging, especially the structural complexity and the relatively small scale of the interface, which averaged from 100 µm to 1 mm in length,^14^,^15^ so traditional biomechanical measurement could not validate the efficacy of the FE model. Further studies are required to validate the efficacy of proposed FE model.
